# Escin Preincubation Enhances the Therapeutic Effect of Umbilical Cord-Derived Mesenchymal Stem Cells in a Rat Model of Myocardial Infarction

**DOI:** 10.1155/sci/1115668

**Published:** 2025-11-21

**Authors:** Xin Yu, Lihong Jiang, Xiaoyu Yang

**Affiliations:** ^1^Faculty of Life Science and Technology, Kunming University of Science and Technology, Kunming 650500, China; ^2^Regenerative Medicine Research Center, The First People's Hospital of Yunnan Province, Kunming 650500, China; ^3^Key Laboratory of Yunnan Provincial Innovative Application of Traditional Chinese Medicine, Kunming 650500, China

**Keywords:** cardiac function, escin, myocardial infarction, transcriptomics, umbilical cord-derived mesenchymal stem cells (UCMSCs)

## Abstract

**Introduction:**

Umbilical cord-derived mesenchymal stem cells (UCMSCs) are promising candidates for the treatment of myocardial infarction (MI). However, their low mobility and survival limit their clinical applicability. This study aimed to enhance the therapeutic potential of UCMSCs by preincubating them with escin, a natural medicine derived from the dried mature seeds of *Aesculus wilsonii*.

**Methods:**

We characterized the functional properties of UCMSCs before and after escin preconditioning in vitro. Additionally, we performed RNA sequencing (RNA-seq) to analyze the transcriptomic differences between untreated and escin-pretreated UCMSCs (E-UCMSCs), followed by Western blot (WB) validation of the differentially expressed genes. In vivo, an MI model was established in rats, which involved permanent ligation of the left anterior descending coronary artery, followed by intravenous administration of UCMSCs and E-UCMSCs through the tail vein. The therapeutic efficacy of UCMSCs and E-UCMSCs was assessed by cardiac function measurements and Masson's trichrome staining to quantify fibrosis.

**Results:**

No significant differences were observed in the basic characteristics of the UCMSCs before and after escin pretreatment. RNA-seq results demonstrated higher expression of intercellular adhesion molecule 1 (ICAM1) and GATA-binding protein 4 (GATA4) in E-UCMSCs than in UCMSCs. Furthermore, WB results confirmed this phenomenon. Most importantly, E-UCMSCs significantly restored myocardial contractile function and reduced infarct size in MI rats.

**Conclusions:**

The current study demonstrates that escin upregulated ICAM1 and GATA4 gene expression in UCMSCs, thereby enhancing the therapeutic efficacy of UCMSCs in rats with MI. Therefore, pretreatment of UCMSCs with escin is a promising approach for the treatment of MI.

## 1. Introduction

Myocardial infarction (MI) is a leading cause of global morbidity and mortality, contributing to approximately 7 million deaths annually [1, 2]. It is primarily due to severe ischemia and hypoxia in the heart tissue caused by the reduction or obstruction of coronary blood flow, which aggravates the inflammatory response and leads to a significant loss of cardiomyocytes [3]. Inflammation is a pivotal pathogenic factor in MI, and excessive and chronic postinfarction inflammation ultimately leads to heart failure [4]. Currently, clinical treatments for MI include thrombolytic medications, percutaneous coronary intervention, and coronary artery bypass grafting [5, 6]. However, these treatments are unable to resolve the inflammatory response or induce tissue repair and are less effective in patients with severe MI and heart failure [6, 7]. Therefore, effective therapeutic strategies are required to suppress inflammatory responses and restore myocardial function after MI.

Recently, stem cell therapy has emerged as a promising therapeutic approach for MI because it shows potential in regenerating damaged cardiac tissue [8–10]. Umbilical cord-derived mesenchymal stem cells (UCMSCs), which are characterized by their high proliferative capacity, multilineage differentiation potential, low immunogenicity, and diverse biological functions, have been widely investigated for cardiovascular repair, particularly for alleviating myocardial damage following infarction [11–14]. The immunosuppressive ability and potential differentiation of UCMSCs into cardiomyocyte-like cells have attracted considerable attention. However, UCMSCs transplantation for tissue regeneration faces multiple obstacles, including low cell retention rates and variable immunosuppression results. Consequently, developing effective UCMSCs-based therapies for MI remains a critical challenge.

Escin, a triterpene saponin with ester bonds, is derived from desiccated, fully developed seeds of *Aesculus hippocastanum* [15]. Studies have shown that escin has anti-inflammatory, detumescent, and neuroprotective properties [16–18]. Qiao et al. [19] reported that escin inhibits oxidative stress, apoptosis, and inflammation in cardiomyocytes under hypoxic conditions. Subsequently, Ma et al. [20] found that escin significantly decreased the infarct area and attenuated myocardial damage in a rat model of MI. Thus, escin may be a novel treatment option for MI. However, our understanding of escin's effect on UCMSCs remains limited, and the therapeutic effect of escin-treated UCMSCs on MI remains unclear. This study investigated the therapeutic effects of escin-pretreated UCMSCs (E-UCMSCs) on MI in rats.

In this study, we compared the differences between UCMSCs and E-UCMSCs at the gene level, and their therapeutic effects on MI in rats. Our findings contribute to our understanding of the potential of E-UCMSCs as a therapeutic approach for MI.

## 2. Materials and Methods

### 2.1. Isolation and Culture of UCMSCs

Human umbilical cords (UCs) were aseptically collected with informed consent from the full-term cesarean deliveries of healthy pregnant women at the First People's Hospital of Yunnan Province, Kunming, China. The experimental protocols of this study were approved by the Institutional Ethics Committee (Approval Number: KHLL2022-KY155) of the First People's Hospital of Yunnan Province. Fresh UCs were stored in sterile physiological saline (0.9% NaCl). Blood vessels (arteries and veins) were meticulously removed, and Wharton's jelly tissue was dissected into 1–3 mm^3^ explants. Explants were evenly distributed in 10 cm culture dishes and maintained in mesenchymal stem cell (MSC)-specific serum-free medium (MSC-T4, CSTI, Japan) supplemented with 5% UltraGRO (Helios, USA). Primary cells (P0) migrated from explants after 10–15 days under standard conditions (37°C, 5% CO_2_). P0 UCMSCs were detached using CTS TrypLE Select (Gibco, USA), passaged at 80% confluence, and cultured to passage 3 (P3). The P3 cells were cryopreserved in liquid nitrogen for subsequent experiments.

### 2.2. Cell Viability Assay

UCMSCs were seeded in 96-well plates at a density of 3000 cells/well and cultured under standard conditions (37°C, 5% CO_2_). After 24 h of adhesion, cells were treated with varying concentrations of escin (MCE, USA) in a final volume of 100 μL/well. Following 72 h of incubation (37°C, 5% CO_2_), 10 μL of Cell Counting Kit-8 (CCK-8) reagent (MCE) was added to each well. After 2–4 h of incubation, the absorbance was measured at 450 nm using a microplate reader (Allsheng, China).

### 2.3. Cell Cycle Assay

This assay was performed using a DNA content detection kit (Solarbio, China). UCMSCs were pretreated with 10 μM escin for 72 h, trypsinized (Gibco), and centrifuged (400 × *g*, 5 min). The cell pellet was washed twice with 0.9% NaCl, then fixed in 500 μL of ice-cold 70% ethanol overnight at 4°C. The following day, fixed cells were washed with 0.9% NaCl to remove ethanol, resuspended in 100 μL of RNase A solution, and incubated at 37°C for 30 min to digest RNA. Subsequently, 400 μL of propidium iodide (PI) staining solution was added, and cells were incubated in the dark at 4°C for 30 min. Cell cycle distribution was analyzed using a flow cytometer (Agilent, USA) with excitation at 488 nm.

### 2.4. Flow Cytometric Analysis of UCMSCs Surface Markers

Following a 72 h pretreatment with escin (10 μM), UCMSCs were harvested and stained with the fluorochrome-conjugated antibodies listed below (all from BioLegend, USA) for surface marker characterization. The positive MSCs markers included CD44-FITC (Cat: B285079), CD90-PE (Cat: 355412), CD105-APC (Cat: B337814), and CD73-APC/Cyanine 7 (Cat: B355163). Negative MSCs markers included: CD19-PerCP (Cat: B285536), CD45-FITC (Cat: B354432), CD34-PE (Cat: B320635), CD11b-APC (Cat: B278346), and HLA-DR-APC/Cyanine 7 (Cat: B353658). Cells were incubated with 5 μL of each antibody (or isotype control) for 30 min at room temperature in the dark. The cells were washed thrice with 0.9% NaCl to remove unbound antibodies. Samples were resuspended in 200 μL 0.9% NaCl and analyzed using a flow cytometer (Agilent, USA).

### 2.5. RNA Sequencing (RNA-seq)

Following a 72 h pretreatment with escin (10 μM), total RNA was extracted from UCMSCs using the RNAiso Plus reagent (Tiangen Biotech, China) according to the manufacturer's protocol. Sequencing was performed by Illumina NovaSeq 6000 (Guangzhou Gene Denovo Biotechnology Co., China). Gene expression was quantified using featureCounts, and differential expression analysis was conducted using the DESeq2 (4) software. Differentially expressed genes were defined as those with a |log_2_(fold change)| ≥ 1 and an adjusted *p*-value (FDR) < 0.05. The Kyoto Encyclopedia of Genes and Genomes (KEGG) pathway analysis was performed using the clusterProfiler R package.

### 2.6. Western Blot (WB) Analysis

After 72 h of escin (10 μM) pretreatment, UCMSCs were harvested and lysed in RIPA buffer (Beyotime, China) on ice for 30 min. Protein concentration was determined using the Bradford assay (Beyotime, China). Equal amounts of protein (20 μg per lane) were separated by 10% sodium dodecyl sulfate-polyacrylamide gel electrophoresis (SDS-PAGE), using a Beyotime kit, and transferred onto nitrocellulose membranes (0.45 μm pore size) via wet transfer. Membranes were blocked with 5% nonfat milk (Wandashan, China) for 1 h at room temperature and were incubated overnight at 4°C with antibodies targeting ICAM1 (AF1774, Beyotime), GATA4 (ab256782, Abcam, USA), and GAPDH (AF7021, Affinity, USA). After washing, membranes were incubated with HRP-conjugated secondary antibodies (ab6721, Abcam) for 1 h at 20°C. The protein bands were visualized using an enhanced chemiluminescence (ECL) substrate (MCE, USA).

### 2.7. Animal Experiment

Male Sprague-Dawley (SD) rats (5–6 weeks old) were purchased from Beijing Vital River Laboratory Animal Technology Co., Ltd., and housed under standard laboratory conditions (temperature: 25°C; relative humidity: 55%–60%). All animal procedures were approved by the Institutional Animal Ethics and Welfare Committee of MDKN Biotechnology Co., Ltd. (Approval Number.: MDKN-2024-090). Under anesthesia, permanent ligation of the left anterior descending coronary artery was performed to induce MI. Two weeks post-MI, rats were randomly divided into the following treatment groups (*n* = 4/group): (1) MI+NS (negative control): 500 μL 0.9% NaCl (intravenous, tail vein); (2) MI+UCMSCs: 1 × 10^6^ UCMSCs in 500 μL 0.9% NaCl (intravenous, tail vein); and (3) MI+E-UCMSCs: 1 × 10^6^ E-UCMSCs in 500 μL 0.9% NaCl (intravenous, tail vein).

At 21 days postcell therapy, transthoracic echocardiography was performed on all rats under anesthesia. Left ventricular parameters were measured in the parasternal long-axis view. The echocardiography system automatically calculated the left ventricular ejection fraction (LVEF) and left ventricular fractional shortening (LVFS) to assess cardiac function. Following echocardiography, the rats were humanely euthanized, and heart tissues were harvested. Excised tissues were immediately fixed in 4% paraformaldehyde for 24 h at 4°C for subsequent histological analysis.

### 2.8. Masson's Trichrome Staining

Fixed heart tissues were embedded in paraffin, and each heart was sectioned transversely into five levels from the base to the apex. At each level, 5 μm-thick sections were prepared using a microtome (Leica, Germany). Sections were stained with Masson's trichrome solution, according to the manufacturer's protocol, to differentiate collagen fibers (blue) from viable myocardium (red). The stained slides were digitally scanned using a Pannoramic MIDI scanner (3D HISTECH). The fibrotic area was quantified using ImageJ software.

### 2.9. Immunofluorescence Staining

Fixed heart tissues were embedded in paraffin, and each heart was sectioned transversely into five levels from the base to the apex. At each level, 5 μm-thick sections were prepared using a microtome (Leica, Germany). The sections were dewaxed with xylene and ethanol, soaked in citric acid antigen repair buffer (pH 6.0, G1202, Servicebio), and heated in a microwave oven for antigen repair. After blocking with bovine serum albumin (GC305010, Servicebio) at room temperature for 30 min, the sections were incubated overnight at 4°C with primary antibody (CD31: GB113151, Servicebio; Ms X Hu Nuclei antibody: MAB1281, Merck), washed, and incubated with secondary antibody (GB21303, Servicebio) for 2 h at room temperature. The nuclei were stained with DAPI (G1012, Servicebio) after washing and incubated in the dark at room temperature for 10 min. Finally, the stained sections were observed using a microscope.

### 2.10. PBMCs Proliferation Assay

To evaluate the immunosuppressive capacity of E-UCMSCs, we established a coculture system where 2 × 10^5^ UCMSCs were seeded per well in six-well plates overnight, followed by the addition of 1 × 10^6^ CFSE (150347-59-4, TargetMol)-labeled peripheral blood mononuclear cells (PBMCs, CP-H158, Procell) and 5 μg/mL phytohemagglutinin (PHA, 40110ES08, YEASEN) for 72 h at 37°C with 5% CO_2_. Following incubation, PBMCs were collected, and their proliferation was analyzed by flow cytometry through measurement of CFSE dye dilution. The percentage of divided cells and proliferation index were calculated using FlowJo software.

## 3. Results

### 3.1. The Characterization of E-UCMSCs Was Consistent With That of UCMSCs

**Figure 1:**
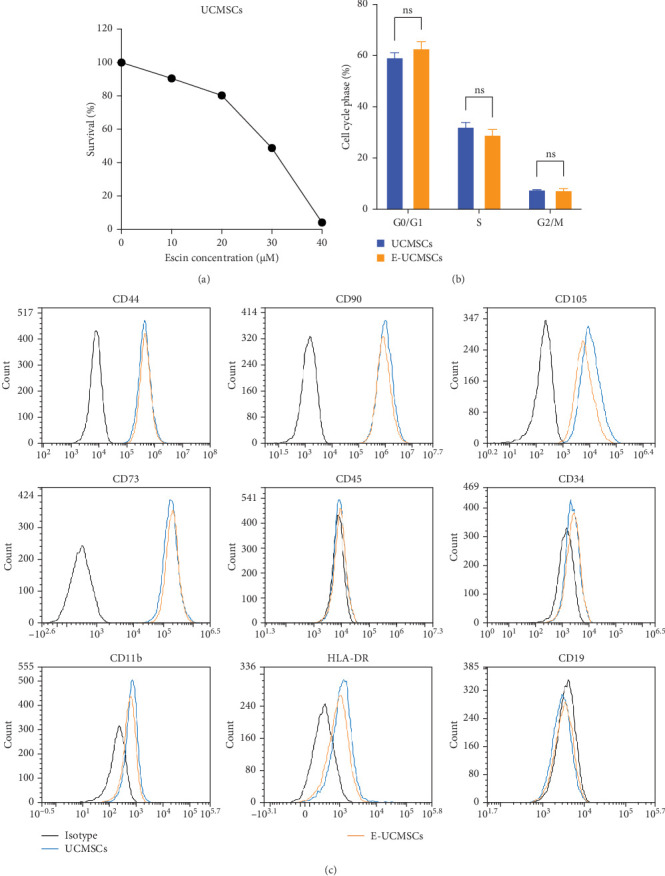
The characterization of E-UCMSCs. (a) CCK-8 was used to detect the activity of UCMSCs after treatment with different concentrations of escin at 72 h. *N* = 5. (b) Cell cycle distribution of UCMSCs after escin treatment. *N* = 3. Error bars, mean ± S.E.M., unpaired two-tailed *t*-test. (c) Surface marker profiling of UCMSCs after escin treatment. The black lines represent the isotype, the blue lines represent corresponding CD marker expression in UCMSCs, and the orange lines represent corresponding CD marker expression in E-UCMSCs. ns, not significant.

UCMSCs were successfully isolated and cultured from the human UCs. To determine the optimal escin concentration, we treated UCMSCs with different concentrations of escin and then conducted cell viability assays. The results showed that the concentration of escin at 10 μM had only a small effect on UCMSCs viability (Figure 1a). Moreover, escin at the concentration of 10 μM had no significant effect on the cell cycle of UCMSCs (Figure 1b). Flow cytometry confirmed that E-UCMSCs also express specific mesenchymal cell markers, including CD44, CD73, CD90, and CD105 (≥95%), but lack CD19, CD34, CD45, CD11b, and HLA-DR (≤2%; Figure 1c). These results indicated that escin has no significant effect on the basic characteristics of UCMSCs.

### 3.2. E-UCMSCs Expressed Relatively High Levels of Intercellular Adhesion Molecule 1 (ICAM1)

**Figure 2:**
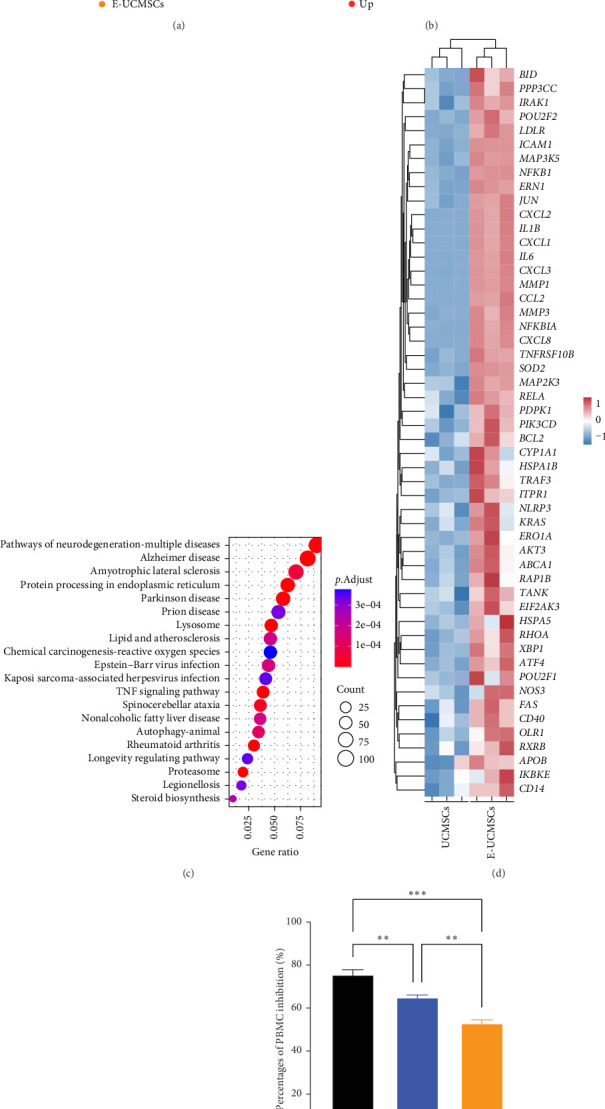
E-UCMSCs express relatively high levels of ICAM1. (a) PCA cluster analysis showed statistically significant differences among the groups. (b) Volcano map of UCMSCs vs. E-UCMSCs. (c) KEGG pathway enrichment analysis (top 20) of upregulated genes in E-UCMSCs. (d) Heatmap of gene expression in the lipid and atherosclerosis pathway. (e) Western blot was used to detect the expression levels of ICAM1 in E-UCMSCs. (f) In vitro PBMC proliferation assay demonstrating that E-UCMSCs exhibit stronger suppression of immune cell proliferation compared to UCMSCs. *N* = 3. Error bars, mean ± S.E.M., one-way ANOVA. *⁣*^*∗∗*^*p* < 0.01, *⁣*^*∗∗∗*^*p* < 0.001.

To investigate the gene-level changes induced by escin in UCMSCs, we analyzed the transcriptome and gene expression in E-UCMSCs using RNA-seq technology. Principal component analysis (PCA) revealed a shift in principal components (PC) 1 and PC2 in E-UCMSCs compared to UCMSCs (Figure 2a). Compared to UCMSCs, there were 2224 upregulated and 2089 downregulated genes in E-UCMSCs (Figure 2b). Subsequently, KEGG pathway enrichment analysis (top 20) was performed on the upregulated genes in E-UCMSCs (Figure 2c). Among them, the lipid and atherosclerosis pathway, which is related to MI, was analyzed further. There were 52 upregulated genes related to the lipid and atherosclerosis pathway, including ICAM1, a key player in MSCs-mediated immunosuppression (Figure 2d). Several studies have shown that MSCs overexpressing ICAM1 have an enhanced immunosuppressive capacity [21, 22]. WB analysis confirmed that E-UCMSCs exhibited higher ICAM1 expression levels compared to conventional UCMSCs (Figure 2e). This observation was consistently reproduced in another UCMSCs line derived from a different maternal source (Figure S1A), demonstrating that the elevated ICAM1 expression in E-UCMSCs is not a stochastic event but represents a reproducible characteristic. Subsequent in vitro experiments revealed a more potent immunosuppressive capacity of E-UCMSCs, as evidenced by their significantly enhanced inhibition of PBMCs proliferation relative to their non-engineered counterparts (Figure 2f). Thus, escin can induce high ICAM1 expression in UCMSCs, which may increase their immunosuppressive ability. Overall, these results indicate that escin induces dramatic changes in UCMSCs at the gene level and that E-UCMSCs may have a higher immunosuppressive capacity, and therefore show greater inhibition of the inflammatory response due to high expression of ICAM1, thereby enhancing the therapeutic effect against MI-induced damage.

### 3.3. E-UCMSCs Expressed Relatively High Levels of GATA4

**Figure 3:**
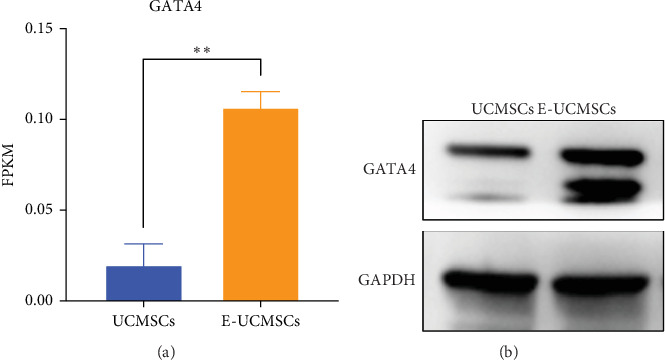
E-UCMSCs express relatively high levels of GATA4. (a) FPKM of GATA4 in E-UCMSCs. *N* = 3. Error bars, mean ± S.E.M., unpaired two-tailed *t* test. (b) Western blot was used to detect the expression levels of GATA4 in E-UCMSCs. *⁣*^*∗∗*^*p* < 0.01.

Our previous study demonstrated that maternal segment-derived MSCs, which highly express GATA4 (a key transcription factor in cardiomyocyte development), exhibit superior efficacy in attenuating MI in rats [23]. To investigate whether escin preconditioning similarly enhanced GATA4 expression, we analyzed the RNA-seq data and found that E-UCMSCs showed significantly higher fragments per kilobase of transcript per million reads mapped (FPKM) values for GATA4 than that of UCMSCs (Figure 3a). Further validation by WB confirmed that GATA4 protein expression was markedly upregulated in E-UCMSCs compared to that in untreated UCMSCs (Figure 3b). These results suggest that escin preconditioning induces GATA4 overexpression in UCMSCs, which may contribute to their enhanced cardioprotective potential.

### 3.4. E-UCMSCs Effectively Protect Cardiac Function in Rats with MI

**Figure 4:**
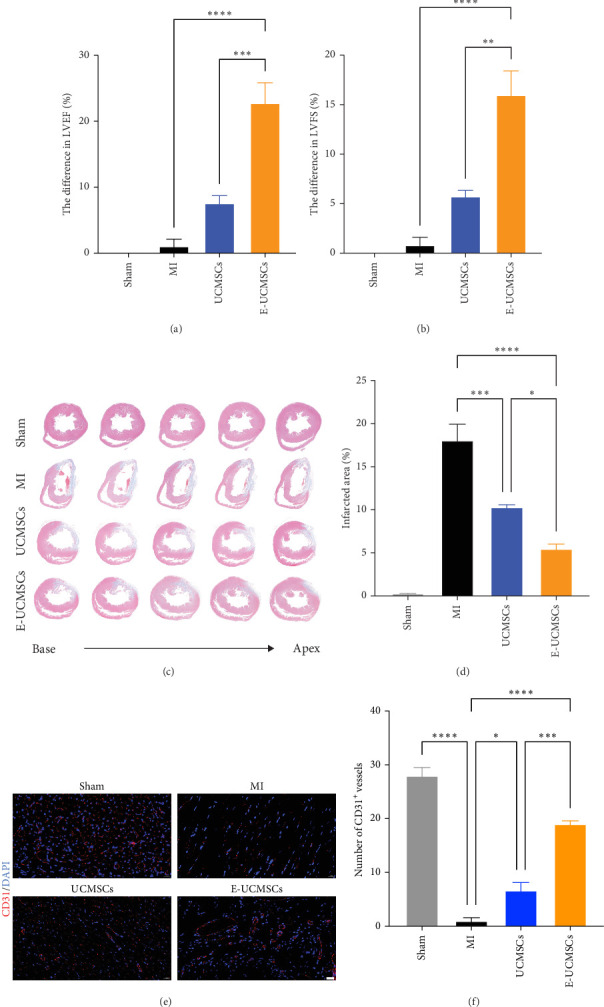
E-UCMSCs effectively protected the cardiac function of rats with MI. (a,b) Quantitative analysis of the differences in LVEF and LVFS before and after cell therapy. *N* = 4. Error bars, mean ± S.E.M., one-way ANOVA. (c) Representative Masson's trichrome staining of five regions from the base to the apex of the infarcted heart after cell treatment. Blue indicates fibrotic tissue, and red indicates normal myocardial tissue. (d) Quantitative analysis of the infarct size. *N* = 4. Error bars, mean ± S.E.M., one-way ANOVA. (e) Representative image showing immunofluorescent staining for CD31 (red) in the hearts of rats with MI in different groups after treatment. Nuclei are stained blue with DAPI. Scale bar = 20 μm. (f) Quantitative analysis of blood vessels stained with CD31. *N* = 3. Error bars, mean ± S.E.M., one-way ANOVA. *⁣*^*∗*^*p* < 0.05, *⁣*^*∗∗*^*p* < 0.01, *⁣*^*∗∗∗*^*p* < 0.001, *⁣*^*∗∗∗∗*^*p* < 0.0001.

To investigate the therapeutic effects of E-UCMSCs, we established a rat MI model induced permanent ligation of the left anterior descending coronary artery followed by intravenous E-UCMSCs transplantation through the tail vein. At 21 days post-MI, transthoracic echocardiography was performed to evaluate the therapeutic efficacy of E-UCMSCs (Figure S1B). Statistical analysis of the differences in LVEF and LVFS before and after treatment revealed that UCMSCs treatment significantly attenuated the MI-induced decline in LVEF and LVFS (Figure 4a,b). Notably, the improvements in LVEF and LVFS were markedly greater in the E-UCMSCs group than in the UCMSCs group (Figure 4a,b), suggesting that escin preconditioning enhances the cardioprotective effects of UCMSCs. To assess the extent of MI-associated fibrosis, Masson's trichrome staining was performed 21 days after UCMSCs transplantation (Figure 4c). Quantitative analysis revealed that the fibrotic area was significantly reduced in the E-UCMSCs group compared to that of the UCMSCs group (Figure 4d), indicating that E-UCMSCs effectively ameliorated cardiac fibrosis. To further evaluate the cardioprotective effects of E-UCMSCs, we assessed vascular integrity in infarcted hearts using CD31 immunofluorescence staining. Immunofluorescence analysis revealed a significant increase in capillary formation in E-UCMSCs-treated hearts compared to the UCMSCs group (Figure 4e,f). Collectively, these findings provide additional evidence that E-UCMSCs confer significant improvement in cardiac functional recovery.

Furthermore, to elucidate the potential mechanisms by which E-UCMSCs ameliorate the infarcted heart, we used a human-specific nuclear antibody to detect transplanted human cells in rat hearts after 21 days. Positive signals were found in the E-UCMSCs group, but not in the untreated group (Figure S1C). These results suggest that escin pretreatment enhances the retention or survival of MSCs under ischemic conditions. Although further studies are needed to clarify the underlying mechanism, these findings indicate a potential way escin may improve therapeutic outcomes—a focus of our future work.

## 4. Discussion

MI poses a significant challenge because of its deleterious effects on cardiac function [24]. Despite ongoing research into novel therapeutic avenues, there is a lack of effective strategies that enhance myocardial repair and functional recovery [25]. In this study, we investigated the effects of escin on UCMSCs and sequenced the transcriptome of E-UCMSCs for the first time. The results showed that ICAM1 (adhesion molecule) and GATA4 (cardiac transcription factor) were highly expressed in the E-UCMSCs. Subsequently, E-UCMSCs were used to treat MI in rats. As expected, E-UCMSCs promoted recovery of cardiac function and reduced infarct size.

MSCs are derived from bone marrow, adipose tissue, endometrium, UCs, and the amniotic membrane, among others. UCMSCs, due to their easy and minimally invasive access, multilineage potential, immunomodulatory capacity, and minimal ethical concerns, have been widely used in preclinical and clinical studies [26, 27]. Here, we used transcriptome sequencing to determine the differences in gene expression between E-UCMSCs and UCMSCs. Our RNA-seq results showed that E-UCMSCs had gene expression patterns that were significantly different from those of UCMSCs. Importantly, the expression of ICAM1, a gene associated with cell adhesion, and GATA4, a gene involved in cardiomyocyte development, were both significantly upregulated in E-UCMSCs. These results are consistent with previous studies that have identified several factors that enhance the immunomodulatory and therapeutic functions of UCMSCs [28]. de Witte et al. [29] showed that the pretreatment of UCMSCs with cytokines in vitro resulted in decreased immunogenicity, increased immunomodulatory capacity, and enhanced efficacy in ameliorating liver inflammation. Furthermore, Sahibdad et al. [30] showed that the addition of low concentrations of Zn in vitro enhanced UCMSCs migration and adhesion. Therefore, in vitro pretreatment of UCMSCs has the potential to enhance the therapeutic effects of UCMSCs.

In this study, we pretreated UCMSCs with escin in vitro and found that E-UCMSCs exhibited significantly higher expression of ICAM1 and GATA4 than untreated controls. Based on these findings, we hypothesized that E-UCMSCs exert superior cardioprotective effects against MI, which was subsequently validated in a rat model of MI. ICAM1, a member of the immunoglobulin superfamily of cell adhesion molecules, plays a critical role in intercellular signaling, immune response modulation, and cellular differentiation. It is also closely associated with lymphocyte homing and recirculation [21, 31]. Functionally, ICAM1 enhances the immunosuppressive capacity of MSCs by promoting T cell adhesion [22, 32–35]. Notably, when ICAM1 was functionally blocked or genetically knocked out, the immunosuppressive effects on MSCs were substantially reversed. Increasing the surface adhesion of MSCs, which can be specifically targeted to overexpressed ICAM1 in response to local inflammation, significantly enhances the adhesion of MSCs to the infarcted heart and enhances the treatment of MI [36]. Our findings demonstrate that escin preconditioning markedly upregulates ICAM1 expression in UCMSCs—an effect consistently observed across multiple cell lines. Given the well-established role of ICAM1 in mediating the immunomodulatory functions of MSCs, we propose that its pronounced upregulation represents a key mechanism underlying the enhanced immunosuppressive capacity observed in E-UCMSCs. Although the precise signaling pathways through which escin induces ICAM1 expression require further elucidation, our data establish a strong correlation and provide a plausible mechanistic explanation for this phenomenon. GATA4 is a pivotal transcription factor associated with cardiac development and injury repair [37, 38]; it regulates cell differentiation, proliferation, and survival [39, 40]. Overexpression of GATA4 not only enhances MSCs differentiation but also improves MSCs survival under ischemic conditions, promotes angiogenesis, and ameliorates cardiac function [41, 42].

Although the present study did not elucidate the precise molecular mechanisms by which escin preconditioning enhances UCMSCs function, our data consistently demonstrate that escin pretreatment significantly upregulates ICAM1 expression and augments the immunosuppressive capacity of UCMSCs. Furthermore, in a rat model of MI, transplantation of E-UCMSCs markedly improved cardiac function recovery. These findings provide a solid foundation for future mechanistic investigations. We plan to conduct further studies focusing on the relevant signaling pathways, particularly the role of ICAM1 and its downstream effectors, to unravel the molecular mechanisms through which escin enhances the therapeutic efficacy of UCMSCs.

## 5. Conclusion

In summary, we investigated the differences between E-UCMSCs and UCMSCs. The present study showed that E-UCMSCs highly expressed ICAM1 and GATA4. In addition, E-UCMSCs treatment significantly improved cardiac function and reduced myocardial infarct size in an MI rat model, showing enhanced cardioprotective effects. Characterizing the signaling pathways that mediate these effects remains an avenue for future research. Our results provide compelling evidence supporting the therapeutic potential of escin-treated UCMSCs in attenuating MI injury, and further exploration of its complex molecular mechanisms is warranted.

## Data Availability

The data that support the findings of this study are available from the corresponding author upon reasonable request.

## Nomenclature

GAPDH: Glyceraldehyde-3-phosphate dehydrogenase
GATA4: GATA-binding protein 4
ICAM1: Intercellular adhesion molecule 1
KEGG: Kyoto encyclopedia of genes and genomes
LVEF: Left ventricular ejection fraction
LVFS: Left ventricular fractional shortening
MSCs: Mesenchymal stem cells
MI: Myocardial infarction
PCA: Principal component analysis
PI: Propidium iodide
UCMSCs: Umbilical cord-derived mesenchymal stem cells
UCs: Umbilical cords.

## Supporting Information

Supporting Information Figure S1. E-UCMSCs effectively protected the cardiac function of rats with MI. (A) Western blot was used to detect the expression levels of ICAM1 in E-UCMSCs. (B) Representative echocardiographic images of the hearts of rats with MI in different groups before and after treatment. (C) Representative image showing immunofluorescent staining for CD31 (red) in the hearts of rats with MI in different groups after treatment. Nuclei are stained blue with DAPI. Scale bar = 20 μm.
