# Supplementary figures and images for: Escin Preincubation Enhances the Therapeutic Effect of Umbilical Cord-Derived Mesenchymal Stem Cells in a Rat Model of Myocardial Infarction
Source: Stem Cells Int. 2025 Nov 21;2025:1115668. doi: 10.1155/sci/1115668 (PMC12662690; doi:10.1155/sci/1115668)

A

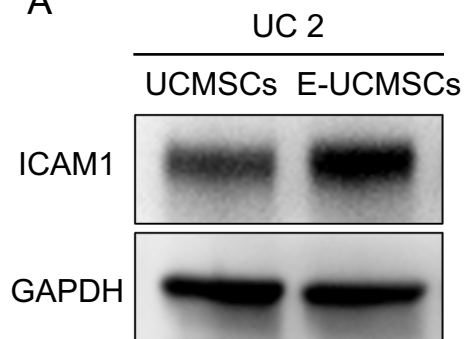

B

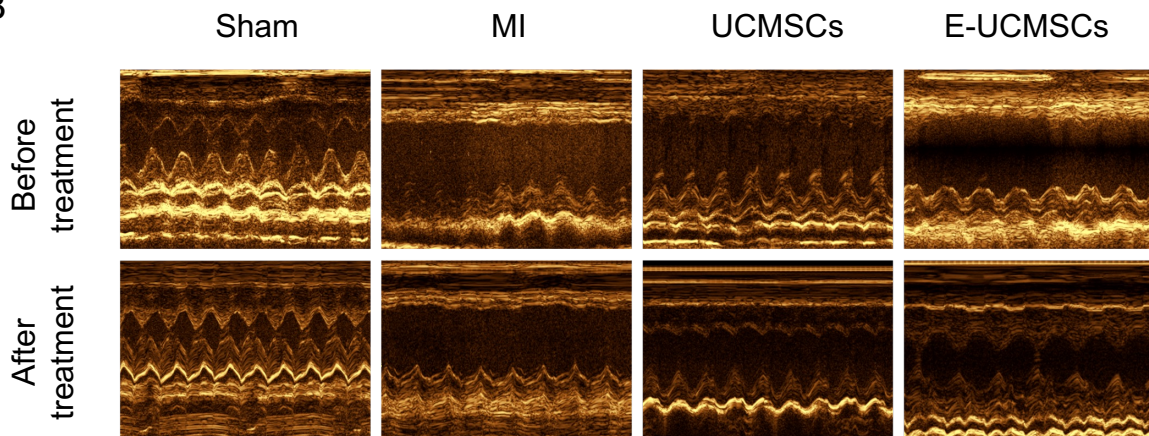

C

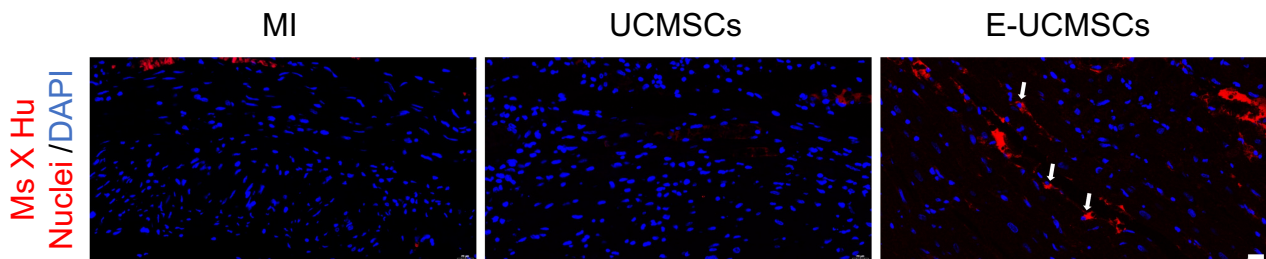

Supplement: Supporting Information — Figure S1. E-UCMSCs effectively protected the cardiac function of rats with MI. (A) Western blot was used to detect the expression levels of ICAM1 in E-UCMSCs. (B) Representative echocardiographic images of the hearts of rats with MI in different groups before and after treatment. (C) Representative image showing immunofluorescent staining for CD31 (red) in the hearts of rats with MI in different groups after treatment. Nuclei are stained blue with DAPI. Scale bar = 20 μm. [file 1115668.f1.pdf]
